# Assessing content validity of learner milestones for decolonial global health education: A modified Delphi study

**DOI:** 10.1371/journal.pgph.0005016

**Published:** 2026-03-17

**Authors:** Jeffrey G. Edwards, Christiana M. Russ, Barnabas Alayande, Jared Gabriel Dela Rosa, Mariam Dogar, Tamara N. Fitzgerald, Priscilla Gonzalez, Ans Irfan, Isaac I. Olufadewa, Eliut Rivera-Segarra, Elisha Siegel, Marta Williams, Leah Ratner

**Affiliations:** 1 Boston Children’s Hospital Department of Pediatrics, Harvard Medical School, Boston, Massachusetts, United States of America; 2 Boston Medical Center Department of Pediatrics, Boston University Chobanian and Avedisian School of Medicine, Boston, Massachusetts, United States of America; 3 Harvard Medical School, Boston, Massachusetts, United States of America; 4 Center for Equity in Global Surgery, University of Global Health Equity, Kigali, Rwanda; 5 College of Medicine, University of the Philippines-Manila, Manila, Philippines; 6 Department of Surgery, Duke University School of Medicine, Durham, North Carolina, United States of America; 7 Keck School of Medicine, University of Southern California, Los Angeles, California, United States of America; 8 Slum and Rural Health Initiative, Ibadan, Oyo State, Nigeria; 9 School of Behavioral and Brain Sciences, Ponce Health Sciences University, Ponce, Puerto Rico; 10 Margaret C. Ryan Global Health Program, Boston Children’s Hospital, Boston, Massachusetts, United States of America; 11 Division of Global Health Equity, Brigham and Women’s Hospital, Boston, Massachusetts, United States of America; PLOS: Public Library of Science, UNITED STATES OF AMERICA

## Abstract

The field of global health is rooted in colonialism, and many global health partnerships perpetuate colonial power dynamics. To change this, knowledge and praxis in decoloniality must become a fundamental part of global health education and practice. Since there are no current tools in global health education specifically focusing on decolonial praxis, we drew on existing literature to develop a tool designed to measure a learner clinician’s progression in attitudes and practice regarding health equity and decoloniality. Milestones were defined across four learner levels: pre-contemplative, contemplative, critical action, and transformative action. Both a self-assessment and an educator assessment of a learner version of the tool were created. We conducted a modified Delphi process using an online survey to create consensus on 20 learner milestone definitions for this assessment tool. To craft a diverse Delphi panel, we invited 11 academic clinicians who have published at least one peer-reviewed publication surrounding decoloniality; six of whom were primarily based in a resource-denied setting, five of whom were primarily based in a resource-advantaged setting, and with at least one participant from sub-Saharan Africa, Central and South America, and Southeast Asia. Consensus was defined as >75% of panelists accepting the definition. After three rounds, Delphi members had reached consensus on all (20/20) items for both the self-assessment and educator assessment versions of the tool. Themes of changes to the tool included better reflecting the mutual learning that occurs between global partners, providing more observable behaviors for assessment, and incorporating transformative learning principles into the milestones, which predominantly utilize a behaviorist framework. This study provides consensus on milestone definitions for a learner assessment tool for clinician trainees in global health that allows learners and educators to engage in self-reflection and progression in decolonial attitudes and practice during an educational process.

## Introduction

Global health (GH) education has come under increasing scrutiny in recent years, particularly within high-income country (HIC) institutions that maintain partnerships with institutions in low- and middle-income countries (LMICs). These partnerships, while often framed as collaborative, frequently perpetuate unequal power dynamics rooted in the colonial history of global health. Traditional notions of global health education are no longer sufficient in addressing the complexities of modern global health practice [1,2]. Colonialism—defined as ‘the practice of extending and maintaining a nations’ political and economic control over another people or area’ has historically shaped the structure and practice of global health [3,4]. As a result, contemporary GH training programs in HICs risk reinforcing inequitable paradigms by centering predominantly HIC-constructed knowledge systems and failing to adequately prepare learners to interrogate their own positionality within these dynamics.

Against this backdrop, efforts to decolonize global health must become not just educational exercises but also acts of resistance and reimagination—demanding personal, institutional, and structural transformation [6–8]. Global health as a field is grappling with how to re-imagine and alter common practices in global partnerships that perpetuate coloniality and its associated harms – which is described as promoting decoloniality within global health. That said, the concept of “teaching” decoloniality is inherently challenging, given the deeply personal and context-specific nature of decolonial praxis [5]. For example, even when discussing terminology to refer to various regions involved in global health partnerships, individuals attempting to promote decoloniality may use terms that lack specificity and lead to overgeneralization [6]. Recognizing the need for agreed-upon terms for this manuscript to provide clarity, we will use ‘Low and Middle-Income Countries (LMIC)’ interchangeably with ‘materially resource-denied settings of the world,’ though we recognize that both terms carry problematic implications.

Despite increasing calls to incorporate decolonial approaches into GH education, educators lack structured tools to assess learners’ progression in understanding and applying decolonial principles. Often, curricula default to superficial application of diversity or inclusion without personal reflection as a means of critically examining positionality within the systems of power they seek to challenge [6]. Furthermore, existing educational models in HICs tend to emphasize action—such as program implementation or advocacy—without requiring the foundational emotional maturity, reflection, and humility needed to engage meaningfully in decolonial praxis [5,10–12]. To date, no validated tools exist to support educators and learners in HIC settings in assessing attitudes and behaviors tied to decoloniality throughout a learner’s development. Himani Bhakuni and Seye Abimbola [7] underscore the persistence of “epistemic injustice,” where the intellectual contributions of scholars and practitioners from low- and middle-income countries are systematically undervalued and ignored [8]. Addressing these issues requires significant emotional intelligence [9] and deep personal reflection as foundational steps before engaging in other aspects of the praxis cycle, including action [10].

This study builds on prior work proposing a theoretical model for decolonial progression in GH learners [13], and responds to the urgent need for educational tools that reflect this complexity. This study builds on prior work by Ratner et al. [13], which proposed a learner progression model to guide trainees in reflecting on and acting toward equity and decoloniality in global health praxis. That original tool was created to accompany a curriculum for GH learners in HIC settings, grounded in a conceptual framework that emphasizes critical self-reflection and emotional maturity as prerequisites for ethical action. While the model was widely shared and adopted to support GH curricula, it had not yet been studied or validated.

In response to this gap, the present study aimed to assess the content validity of that initial tool using a modified Delphi process. Specifically, we sought to achieve expert consensus on milestone definitions across learner levels, drawing on diverse perspectives (from a geographic and institutional standpoint) to ensure the tool’s conceptual and practical relevance. Our goal was not to create a static endpoint, but to refine a flexible assessment framework that can support learners and educators in high-income settings as they engage with the discomfort, accountability, and transformation required by decolonial praxis. This paper describes that process and presents a version of the tool that has had its content validity assessed as one step toward embedding reflection-driven equity frameworks into GH education.

## Methods

### Ethics statement

This study was determined to be exempt by the Boston Children’s Hospital Institutional Review Board, per protocol IRB-P00044316. Study participants were recruited between January 24^th^, 2023 and February 17^th^, 2023, with all participants completing a written consent form. Additionally, the IRB determined that the protocol met the regulatory requirements necessary to obtain a waiver of authorization.

### Initial learner assessment tool

The tool that we used at the beginning of this study was developed and described in Ratner et al, 2023. Although informed by transformative learning theory [11,12] and decolonial frameworks, the initial tool was not derived through a formal research process. Rather, it served as a practical guide to support reflection and growth among learners engaging with global health equity content. The initial tool mirrored the current competency-based model of medical education in which a behaviorist learning theory is primarily applied via the measurement of observable milestones [13,14]. The present study aims to systematically revise and validate that tool using a modified Delphi methodology. Accordingly, this paper does not detail the development process of the original version but instead focuses on the refinement and consensus-building process undertaken to strengthen its content validity.

### Delphi process

The Delphi process comprised three iterative rounds conducted over seven months (February through September) during 2023. This study adhered to best practices in Delphi methodology and followed the CREDES (Conducting and REporting of DElphi Studies) checklist [15] to ensure methodological rigor and transparency. In the first round, participants were provided with the initial iteration of the learner assessment tools and asked to evaluate each item using the following response categories: Accept, Accept with Revisions, and Reject. Feedback was collected anonymously and confidentially through the online survey platform REDCap (Vanderbilt University).

Consensus was defined a priori, based on existing literature [16,17], as 75% or more of the responses falling into the ‘Accept’ category for each item on the assessment tool. The 75% consensus threshold felt appropriate for this study given the smaller panel size of 11, meaning that most participants would need to agree on the milestone definitions, while also allowing for expected discordance amongst such an intentionally heterogenous group. Items that did not meet this threshold were revised according to the feedback provided and included in the subsequent round for re-evaluation. The study was designed to continue until consensus was reached on all items, ensuring that the final tool reflected the collective insights of the participants.

### Defining “Experts”

Experts were defined as individuals who had previously published scholarly work related to global health and decoloniality. The sources of scholarly work included peer-reviewed publications and other forms of scholarship, such as published scholarship and grey literature to broaden inclusion. We utilized PubMed, Web of Science, JSTOR, and Google Scholar research databases, utilizing key terms, such as “decoloniality”, “decolonization”, “global health equity”, “global health”, “global partnership”, “equitable partnerships” and other related terms to identify potential participants. We further utilized citation tracking [18] to identify additional papers that were not initially included in our literature search, including grey literature that was referenced in publications from our initial search. The term “experts” is used with the recognition that no one can truly master this practice and that a core component of decoloniality is the recognition of one’s lifelong commitment to becoming transformative learners. Nevertheless, the Delphi method was employed to achieve consensus among a panel of experts regarding the assessment of learner levels to establish content validity for the tool and to adhere to the Delphi methodological rigor.

### Participant selection

Experts were recruited to participate in the Delphi process through purposive sampling techniques to maintain a group encompassing diverse demographics and perspectives relevant to global health and decoloniality. Participants were selected based on their primary institution’s setting, academic background, and clinical training status, aiming for representation across diverse demographics especially as it related to institutional and geographic diversity. We mandated that at least one Delphi participant was based at an institution in each of the following historically colonized settings (sub-Saharan Africa, the Caribbean or South America, and Southeast Asia) at the time of study recruitment, with our final group containing two members from each of these groups. We additionally mandated that at least one Delphi member have a background in the nursing discipline and that at least one Delphi member was actively in training. Participants were identified through a traditional and grey literature search by authors, who then categorized each Delphi candidate based on institution and training background to meet the target characteristics previously mentioned. Through direct outreach to those candidates (occasionally assisted via professional network connections), we ultimately reached out to 24 individuals, 12 of whom confirmed participation, and 11 who participated throughout the entirety of the study (one participant discontinued participation before the initial Delphi round due to unforeseen circumstances). The final Delphi group was slightly skewed toward individuals from low- and middle-income countries, with six from such institutions and five from institutions in high-income countries (Table 1).

**Table 1:** Delphi member geographic summary. A table detailing the geographic distribution of the modified Delphi members for this study, by the region affiliated with each individual at the time of participation. Regions separated into the following groups: sub-Saharan Africa, Caribbean and South America, Southeast Asia, and Resource-Advantaged (United States, Canada, United Kingdom, and Australia).

Delphi Participant	Region
Participant #1	Sub-Saharan Africa
Participant #2	Sub-Saharan Africa
Participant #3	Southeast Asia
Participant #4	Southeast Asia
Participant #5	Caribbean and South America
Participant #6	Caribbean and South America
Participant #7*	USA, UK, Canada, Australia
Participant #8^	USA, UK, Canada, Australia
Participant #9	USA, UK, Canada, Australia
Participant #10	USA, UK, Canada, Australia
Participant #11	USA, UK, Canada, Australia

* Participant has a background in the nursing discipline.

^ Participant was a trainee at the time of the study.

### Data analysis

JGE and ES analyzed results from each round and presented deidentified data to the initial research team (LR, CMR, and IO). For milestone definitions that lacked consensus, we analyzed response comments and, where applicable, revised items to reflect modal suggestions for changes that would increase the likelihood of consensus in the next round. We presented the quantitative results (percentage for accept, accept with revisions and reject) and de-identified comments (to incorporate qualitative response) to the participants for rounds 2 and 3. Participants were asked to review each learner milestone definition and reconsider their previous responses on the basis of the modifications and/or review of shared data. Participants who did not agree with the response category in rounds 2 and 3 were again prompted to provide further explanations, and these explanations were used to refine the milestone definition and move toward consensus.

### Including Delphi participants as authors

Given the nature of this study and the substantial contributions of the Delphi members to the development of the current set of learner milestones, our research team invited all members of the Delphi team to participate in the authorship of the study manuscript. This practice mirrored the process of self-reflection and careful consideration of one’s actions in the context of the colonial history of global health that we aim to encourage with the product of this study. All members were invited to participate in authorship, though only a subset (five out of eleven) opted in to do so. This process was only initiated after the three rounds of the study had been completed to maintain the anonymity required for an effective modified Delphi study design.

## Results

Our response rate for each round was 100% (n = 11). Consensus was successfully achieved after three rounds of the Delphi process, culminating in the endorsement of the final learner assessment tool by the expert panel. This tool reflects a high degree of agreement among participants and incorporates significant refinements from its initial version, ensuring it serves as a framework with content validity for both self-assessment and faculty assessment of learners. Over the course of the three rounds, consensus was reached on 6 out of 20 items in the first round, 10 out of 20 items in the second round, and 20 out of 20 items in the third round (Table 2). This progressive alignment demonstrates the effectiveness of the iterative process in refining the tool to meet the collective insights and expectations of the expert panel.

**Table 2:** Consensus evolution across Delphi rounds. Demonstration of the Delphi results by round for each domain and learner level. Number indicates the percentage of Delphi participants who voted Accept during that round (n = 11 for every round). Green shaded background indicative of description that met the consensus criteria (>75% of participants voting Accept).

Domain	Domain Level	Round 1	Round 2	Round 3
**Foundational Principles of Equity, Anti-Racism and Decoloniality**	Pre-Contemplative	72.70%	72.70%	90.90%
	Contemplative	90.90%	72.70%	90.90%
	Critical Action	63.60%	81.80%	90.90%
	Transformative Action	63.60%	63.60%	90.90%
**Education**	Pre-Contemplative	81.80%	81.80%	90.90%
	Contemplative	90.90%	72.70%	90.90%
	Critical Action	72.70%	54.50%	90.90%
	Transformative Action	63.60%	90.90%	90.90%
**Research**	Pre-Contemplative	72.70%	54.50%	90.90%
	Contemplative	63.60%	81.80%	90.90%
	Critical Action	81.80%	81.80%	90.90%
	Transformative Action	54.50%	54.50%	90.90%
**Structural Humility**	Pre-Contemplative	54.50%	81.80%	90.90%
	Contemplative	72.70%	81.80%	90.90%
	Critical Action	45.40%	72.70%	90.90%
	Transformative Action	63.60%	72.70%	90.90%
**Leadership and Development**	Pre-Contemplative	63.60%	81.80%	90.90%
	Contemplative	81.80%	90.90%	90.90%
	Critical Action	90.90%	90.90%	100.00%
	Transformative Action	63.60%	63.60%	100.00%

**Fig 1:**
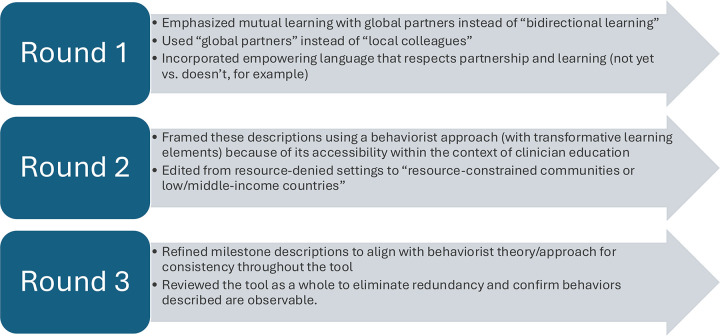
Delphi changes summarized by round. Summary of most frequent and substantive feedback received during each round of the modified Delphi process, with corresponding changes noted.

Major Changes to Milestone Definitions (Fig 1):

1. **Emphasis on Mutual Learning:** One of the most significant revisions was the redefinition of milestones to better reflect the mutual learning that occurs between global partners. This change highlights the importance of bi-directional knowledge exchange, rather than a one-sided transfer of information, aligning with the principles of equity and reciprocity in global health education.
2. **Integration of Observable Behaviors:** To facilitate practical assessment, milestone definitions were refined to emphasize observable behaviors. This adjustment ensures both learners and faculty identify and assess specific actions that demonstrate progression in decoloniality and global health praxis, moving beyond abstract concepts to tangible indicators of learning.
3. **Incorporation of Transformative Learning Principles:** The tool was further enhanced by explicitly integrating transformative learning principles [11,12] into the milestone definitions, rather than relying primarily on a behaviorist framework [14]. This includes recognizing the importance of critical self-reflection, challenging deeply held assumptions, and fostering learner’s capacities to enact change within their professional practice. These principles are now woven throughout the tool, reinforcing the goal of cultivating learners who are not only knowledgeable but also capable of driving systemic change.

**Fig 2:**
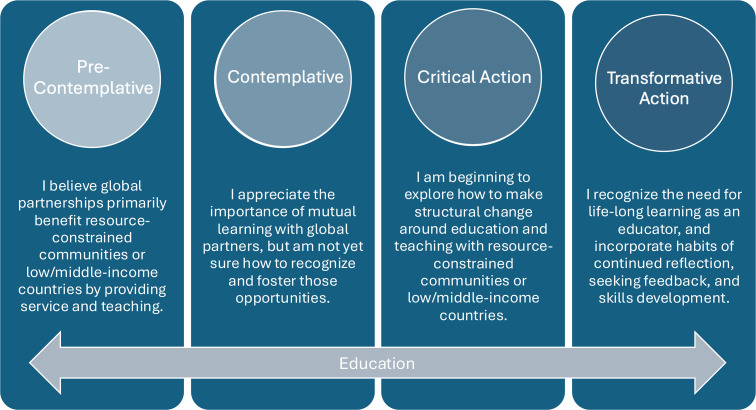
Example of education domain descriptions for self-assessment. Portion of the learner level description for all learner levels within the education domain for the self-assessment version of the tool.

These refinements collectively enhance the utility and applicability of the learner assessment tool, making it a robust resource for guiding the development of learners in health justice and global health towards a deeper engagement with decolonial and equity-based praxis. One example of a revision is for the description for the transformative action learner within the structural humility domain. The initial version stated, “I recognize the differences in cultural and ethical frameworks across settings. I can effectively work with communities and partners to advocate for centering the ethics, values, voices and priorities of communities and promote local leadership. I teach others to recognize the importance of decentering colonial values and ethical frameworks and instead centering local values and priorities.” The consensus version states, “I recognize the importance of respectful engagement of distinct knowledge systems and the importance of iterative self-reflection as I work across settings. I routinely consider my position and privilege in all work with global partners. I can recognize that this work is lifelong while also prioritizing mutual learning with global partners in the communities I work with. I do this to promote individual, structural and systemic change that reflects the contextually derived and locally owned collaboration.” The final consensus reflects a shared commitment to advancing global health education that is both transformational and grounded in the principles of decoloniality. An example of the definitions for the Education domain for all learner levels is demonstrated (Fig 2). The full definitions of each domain at each learner level can be found in both the Self-Assessment (S1 Table) and Educator Assessment of a Learner (S2 Table) forms, respectively.

## Discussion

This study provides the first set of learner milestones with content validity to guide progression in decoloniality and equity within global health education. Utilizing a modified Delphi method, we successfully achieved an a priori-defined consensus of 75% or greater on all 20 learner milestone descriptions for both the self-assessment version and the educator assessment of a learner version. To our knowledge, this is the first study in traditionally published literature to assess the validity of a tool developed for this purpose. These milestones offer a novel framework for educators and trainees—particularly within HIC institutions—to reflect on and advance their engagement with global health justice. Importantly, this work transforms a previously curriculum-bound tool (Ratner et al., 2023) into a rigorously developed, consensus-based assessment instrument.

A key strength of this study lies in its methodological design. The iterative Delphi process allowed for dynamic incorporation of diverse perspectives, including those from historically colonized and resource-denied contexts. Participants brought lived expertise that informed major refinements to the tool—such as the inclusion of observable behaviors and the emphasis on mutual learning and transformative reflection. The high response rate across three rounds and the successful consensus achieved demonstrate both the feasibility and the shared urgency of this work. Central to this approach is the recognition that meaningful action in global health (health justice), particularly in the pursuit of decoloniality and equity, must be rooted in personal reflection and emotional growth. It is not enough to simply identify and act upon issues of coloniality; there must be an intentional process of self-examination that challenges one’s own biases, assumptions, and positionality. This reflective phase is crucial for ensuring that any subsequent actions are genuinely transformative, aligning with the core values of equity and justice.

However, not as a limitation of the study, but rather because of the emotional learning that is required in this field of study-- we caution against using this tool as a prescriptive checklist. The developmental process of decolonial praxis is nonlinear and deeply personal, requiring sustained self-examination, emotional growth, and humility. For these reasons, the milestone definitions are best applied as dynamic guides rather than fixed endpoints. For global health practitioners who operate in the context of academic institutions, we propose that this tool may offer opportunities for growth in decoloniality education and practice while also existing in a familiar framework for those in HIC setting teaching institutions.

### Limitations

The modified Delphi process we employed in this study underscores the importance of interpreting and applying the proposed learner milestones through the lens of a cycle of action, reflection and developing and improving theory - e.g., the praxis cycle [10,19]. However, we recognize that gathering ‘experts’ based on who has written about decolonization in global health will leave out important perspectives, even when seeking to be expansive and include writings outside of academia and representing a range of geographic locations and colonial histories. Although the Delphi process created space for diverse input, it is shaped by structural limitations: participants were selected based on scholarly contributions, a criterion that may inadvertently exclude critical voices, including those without academic access or recognition. Additionally, our study team and participant pool reflect an epistemic imbalance skewed toward individuals trained in biomedical and HIC academic institutions.

We also recognize the tension inherent in using the Delphi method—a consensus-building approach—in a field where complexity, contradiction, and plural truths must be honored. In this context, consensus does not signify an ultimate “truth”, but rather a shared, iterative understanding that is open to further evolution. As such, the tool should be continuously refined as the field and its practitioners grow. No single individual can embody all lived experiences or fully comprehend the diverse realities that shape health justice globally. Therefore, the notion of “expertise” in this context is inherently fluid and contested.

Our goal was that the process was inclusive and reflexive, with a recognition that consensus does not necessarily equate to truth, but rather to a shared understanding that remains open to evolution and further interrogation. Ultimately, the relevance of this work lies in its potential to embed critical self-reflection, accountability, and equity-centered practice into the training of global health professionals. At a time when decolonial discourse in global health has lost momentum—particularly in HIC institutions amid shifting political climates that have reduced funding for multilateral collaboration—this tool offers a concrete, actionable way to sustain that work. By grounding learning in the praxis cycle of reflection, action, and theory-building, these milestones can support more ethically grounded, relational, and equitable approaches to global health practice.

In the end, the question is whether this tool helps move individual global health practitioners and, subsequently, their projects, programs, institutions and systems toward greater equity in global health. In the interim, we strive for greater accountability in how we conduct and present our work. We hope that by openly acknowledging these complexities, we can contribute to a more honest and self-aware discourse on decoloniality in global health education.

## Conclusion

While we, as an author team, believe that the Delphi process and the resulting milestones represent a robust and thoughtful convergence of expert opinion and lived experience, we must emphasize that these tools are not to be used uncritically. The milestones we have developed are intended to guide both educators and learners on their journey toward equity and decoloniality in global health, but they should not be approached as mere checklists. Users must first commit to engaging in the productive discomfort that comes with examining their privilege and positionality. Without this foundational work of self-reflection and humility, the milestones risk being reduced to performative acts rather than transformative steps toward meaningful change. We urge those who utilize these tools to do so with a deep commitment to personal growth and a readiness to confront the complexities and contradictions inherent in decolonial praxis.

## Supporting information

S1 Table Self-assessment. Complete description of each learner level across five domains as a self-assessment, after reaching consensus (>75% agree) in a modified Delphi approach across three rounds. reached in round 1 and re-gained in round 3.
(PDF)

S2 Table Educator assessment of a learner. Complete description of each learner level across five domains as an educator assessment of a learner, after reaching consensus (>75% agree) in a modified Delphi approach across three rounds.
(PDF)

S1 Text Qualitative data excerpts. (DOCX)
