# Supplementary material for: Assessing content validity of learner milestones for decolonial global health education: A modified Delphi study
Source: PLOS Glob Public Health. 2026 Mar 17;6(3):e0005016. doi: 10.1371/journal.pgph.0005016 (PMC12994781; doi:10.1371/journal.pgph.0005016)
Supplement: S1 Table — Complete description of each learner level across five domains as a self-assessment, after reaching consensus (>75% agree) in a modified Delphi approach across three rounds. reached in round 1 and re-gained in round 3. (PDF) [file pgph.0005016.s001.pdf]

	Foundational Principles of Equity, Anti-Racism and Decoloniality	Education	Research	Structural Humility	Leadership and Development
**Pre-Contemplative**	I am unaware of any obvious biases that I hold. I do not believe that my knowledge, views or actions in global health are affected by coloniality or racism.	When teaching in another context or country, I think it is most effective to use the same approach with which I was educated and modifications in teaching are optional. I do not think these teaching strategies could be harmful in other cultures or contexts. Global partnerships primarily benefit resource-constrained communities or low/middle-income countries by providing service and teaching.	I do not see any need to change the current global health research agenda. Research that advances knowledge and health outcomes is inherently beneficial, even if not well-distributed according to local priorities. I believe that funding sources should be allowed to set priorities since they already incorporate community input.	I am aware of how "social determinants" impact health but I don't yet know how society's structures perpetuate inequities in health. I am unaware of how the history of colonialism has a continued impact on the current structures that govern global health. I recognize that there are ethical challenges in global health just as there are in all other fields but I am not sure how different cultures and ethical frameworks impact perceptions of health, wellness, and health care and/or how to consider this in my global health work.	I do not yet understand and/or believe that the history of colonialism still plays a role in development initiatives (economic, environmental, health, etc.) and program management. I do not yet know how power and positionality influence global partnerships and aid. Aid and development are beneficial and I am unaware of how they can be harmful.
**Contemplative**	I am curious as to how and why coloniality and racism exist and manifest themselves in global health. I realize that coloniality and racism are perpetuated by current global health practices, but I'm not sure how to address this in my own work.	I recognize that there are elements of teaching and education that need to be completely modified, or I may need to learn new teaching and education paradigms, based on respecting distinctions within different contexts and cultures. I am curious about how to make these changes when teaching in other contexts. I appreciate the importance of mutual learning with global partners, but am not yet sure how to recognize and foster those opportunities.	I am beginning to recognize the impact of coloniality on global structures and research priority setting with associated harms to marginalized communities. I'm learning how different cultural, ethical and knowledge frameworks impact how healthcare and research are perceived. I am curious about how to effectively prioritize the agendas of global partners and marginalized communities and I am interested in developing the skills to do so.	I am able to recognize the impact of coloniality on global systems. I am able to identify historical and ongoing colonialism but I have not yet identified strategies for working to dismantle these deeply rooted systems. I have started to recognize that there may be different cultural frameworks and values that structure communities and impact how healthcare is perceived and want to develop the skills to incorporate this consideration into my work.	I am starting to recognize the role of coloniality, racism and supremacy in development, and I still struggle with how to make actionable change. I am interested in developing skills to foster local-to-global partnerships that support relational co-learning between global institutions and local communities and organizations. I am curious as to how to identify and include marginalized voices in decision-making processes and leadership.
**Critical Action**	I am actively utilizing decolonial and anti-racist practices in my work (both personally and professionally) at the individual and interpersonal levels. I am learning how to apply these skills more broadly to affect structural change. In collaboration with others, I am working on promoting and teaching these decolonial and antiracist skills and practices.	I respect distinct contexts and cultures in education and make actionable changes around curricula development and delivery based on priorities of my partners, local context, culture, language, and resources. I constantly reflect on how to do this better, critically evaluating my teaching efforts and seeking feedback from learners and global partners. I am working to develop skills for myself and global partners to co-develop effective teaching approaches. I am beginning to explore how to make structural change around education and teaching with resource-constrained communities or low/middle-income countries.	I can apply my knowledge of decoloniality, power, and positionality to promote justice in research practice and research priority setting. I am able to include those who are often marginalized and collaborate with global partners aiming to conduct equitable research. I am still strengthening my skills in fostering mutual learning with global partners and in advocating for systemic changes to promote equity in global health research.	I am curious about my understanding of global health to learn from others with differing worldviews. I can create opportunities for mutual learning with global partners and marginalized communities working within the social reality of culture and colonization. I am learning how to best work within a community's worldview to co-create contextual solutions that promote equity and justice.	I am able to identify and include marginalized voices (as they relate to coloniality, racism, and supremacy) in priority setting, funding allocation and project management. I am curious as to how to support and create policies, systems and structures to support centering marginalized voices and promoting community ownership and leadership more broadly.
**Transformative Action**	Through prior and current work, I am able to share specific examples of applying knowledge of decolonial principles to create structural change across the interpersonal, institutional, community, national and international levels. I am able to advocate with and uplift the voices of global partners through sustainable actions. I am able to help others recognize their role in coloniality and racism, and mentor others in developing their own decoloniality praxis cycles. I am able to promote collective action in decoloniality.	I incorporate local priorities, culture and context into educational initiatives. I effectively center mutual learning with colleagues in resource constrained communities or low/middle income countries in teaching and curricular development and ownership. I advocate alongside global partners, following their lead, to create measurable change in global health education structures and systems, rooted in principles of decoloniality and local ownership that is responsive to the community's needs. I recognize the need for life-long learning as an educator, and incorporate habits of continued reflection, seeking feedback, and skills development.	I work with global partners to promote community voices in all aspects of research generation, including agenda setting, funding and dissemination of research results. I engage in mutual learning and collaboration with global partners in research. Alongside global partners, I actively build research capacity in resource-constrained communities or low/middle-income countries and create systemic change via structures and policies that center community voices. Together, we promote community ownership and authorship of research outputs.	I recognize the importance of respectful engagement of distinct knowledge systems and the importance of iterative self-reflection as I work across settings. I routinely consider my position and privilege in all work with global partners. I can recognize that this work is lifelong while also prioritizing mutual learning with global partners in the communities I work with. I do this to promote individual, structural and systemic change that reflects the contextually derived and locally owned collaboration.	I am able to advocate for decolonial and anti-racist local leadership of programs and funding as they relate to coloniality, racism, and supremacy. I work to actively advance equity, decoloniality and antiracism as well as actively dismantle structures that uphold the persistent oppression, racism and colonialism that marginalize those from resource-constrained communities or low/middle-income countries. I work to collaboratively and actively reimagine and rebuild the practice of global health through a justice-driven lens.
Self-Assessment					
