# Supplementary material for: Assessing content validity of learner milestones for decolonial global health education: A modified Delphi study
Source: PLOS Glob Public Health. 2026 Mar 17;6(3):e0005016. doi: 10.1371/journal.pgph.0005016 (PMC12994781; doi:10.1371/journal.pgph.0005016)
Supplement: S2 Table — Complete description of each learner level across five domains as an educator assessment of a learner, after reaching consensus (>75% agree) in a modified Delphi approach across three rounds. (PDF) [file pgph.0005016.s002.pdf]

	Foundational Principles of Equity, Anti-Racism and Decoloniality	Education	Research	Structural Humility	Leadership and Development
**Pre-Contemplative**	This learner's actions do not convey that they yet recognize their own biases. They have not yet demonstrated awareness that their knowledge, views and actions may be influenced by deeply entrenched colonial and racist systems.	This learner applies the same approach and model of education at their home institution and country to all contexts. They have not yet demonstrated recognition of the potential harm that can come from teaching that overlooks the impact of culture and context. Further this learner has not yet shown awareness of how to engage in mutual learning with global partners, and generally views global exchanges as one-way, benefitting the resource-constrained communities or low/middle-income countries.	This learner has not yet shown consideration for how global health research agendas are set and has not yet openly questioned whether these agendas align with local priorities. They demonstrate a belief that research that advances knowledge and health outcomes is inherently beneficial without asking about local priorities in research or recognizing that they may differ. They may not yet recognize the value of advancing local priorities in research and likely believe that funders are well-equipped to set research agendas and direct research.	This learner may be able to recognize "social determinants" of health but has not yet demonstrated consideration of how structural injustice plays a role in health. They have not yet demonstrated consideration of the impact of the history of colonialism on the structures that govern global health, nor prioritized this in their own learning. This learner recognizes that there are ethical challenges in global health but has not yet shown consideration for those challenges outside of the culture and values in which they were raised and educated. They have not yet articulated how ethical frameworks impact perceptions of health, wellness and health care.	This learner has not yet shown consideration for the persistent impact of colonialism on development initiatives (economic, environmental, health, etc.) and program management. This learner has not yet demonstrated recognition of the potential harms that could come from disempowering local voices. They have not yet shown consideration of the role of power and positionality in global partnerships and aid and tend to view these partnerships and aid solely from the HIC-vantage point and as inherently beneficial.
**Contemplative**	This learner's actions convey curiosity as to how and why coloniality and racism exist and manifest themselves in global health. This is evidenced by how they realize that coloniality and racism are perpetuated by current global health practices and demonstrated by wondering how to address this in their own work.	This learner shows recognition that elements of teaching and education need to be completely modified or that they may need to learn new teaching and education paradigms, based on respecting distinctions within different contexts and cultures. They demonstrate curiosity about how to make these changes in their teaching in other contexts. They demonstrate appreciation for the importance of mutual learning with global partners, but do not yet know how to recognize and foster those opportunities.	This learner has shown initial recognition of the impact of coloniality on global structures and research priority setting with associated harms to marginalized communities. They have started to demonstrate recognition of distinctions in different cultural, ethical and knowledge frameworks that impact how healthcare and research are perceived. They show curiosity about developing skills to prioritize the agenda of global partners and marginalized communities through respectful collaboration.	This learner has demonstrated recognition of the impact of historical and ongoing colonialism on global systems and resulting systemic harms to marginalized communities. They have not yet identified strategies for working to dismantle these deeply rooted structures. They have shown initial recognition that there may be different cultures and values that structure communities and impact how healthcare is perceived and want to develop the skills to incorporate this consideration into their work.	This learner is showing initial recognition of the role of coloniality, racism, and supremacy in development, and demonstrates struggles with how to make actionable change. They show interest in developing skills to foster local-to-global partnerships that support relational co-learning between global institutions and local communities and organizations and demonstrate curiosity as to how to identify and include marginalized voices in decision-making processes and leadership.
**Critical Action**	This learner is actively utilizing decolonial and anti-racist practices in their work (both personally and professionally) at the individual and interpersonal levels. This learner's actions convey that they are still learning how to apply these skills more broadly to affect structural change. In collaboration with others, they are working on promoting and teaching these decolonial and antiracist skills and practices.	This learner shows respect for distinct contexts and cultures in education and makes actionable changes around curricula development and delivery based on priorities of their partners, local context, culture, language, and resources. They are constantly reflecting on how to do this better, critically evaluating their teaching efforts and seeking feedback from learners and global partners. They have shown progress in developing skills for themselves and global partners to co-develop effective teaching approaches. They have demonstrated early exploration of how to make structural change around education and teaching with resource-constrained communities or low/middle-income countries.	This learner has shown application of their knowledge of decoloniality, power, and positionality to promote justice in research practice and research priority setting. They have demonstrated the ability to include those who are often marginalized and collaborate with global partners aiming to conduct equitable research. This learner is still strengthening their skills in fostering mutual learning with global partners and in advocating for systemic changes to promote equity in global health research.	This learner demonstrates recognition that there are differences in understandings of global health which are informed through different worldviews. They have created opportunities for mutual learning with global partners within the social reality of culture and colonization and start to work on an individual level to implement these. They are learning how to best work within a community's worldview to co-create contextual solutions that promote equity and justice.	This learner has identified and included marginalized voices (as they relate to coloniality, racism, and supremacy) in priority setting, funding allocation and project management. They demonstrate curiosity as to how to support and create policies, systems and structures to support centering marginalized voices and promoting community ownership and leadership more broadly.
**Transformative Action**	This learner has demonstrated specific examples of their ability to apply knowledge of decolonial principles to create structural change across the interpersonal, institutional, community, national and international levels. They are able to advocate with and uplift the voices of their global partners through sustainable actions. This learner is able to help others recognize their role in coloniality and racism and mentor others in developing their own decoloniality praxis cycles. They are able to promote collective action in decoloniality.	This learner incorporates local priorities, culture and context into educational initiatives. They have demonstrated effective centering of mutual learning with global partners in resource constrained communities or low/middle-income countries in teaching and curricular development and ownership. They advocate alongside global partners, following their lead, to create measurable change in global health education structures and systems, rooted in principles of decoloniality and local ownership that is responsive to the community's needs. They have shown recognition of the need for life-long learning as an educator, and incorporate habits of continued reflection, seeking feedback, and skills development.	This learner has been observed working with global partners to promote community voices in all aspects of research generation, including agenda setting, funding and dissemination of research results. This learner engages in mutual learning and collaboration with global partners. They work with global partners to actively build research capacity in resource-constrained communities or low/middle-income countries and create structures and policies that support centering community voices. Alongside global partners, they promote community ownership and authorship of research outputs.	This learner demonstrates recognition of the importance of respectful engagement of distinct knowledge systems and importance of lifelong iterative self-reflection across settings. They have shown consideration of their position and privilege in all work with global partners. They prioritize mutual learning with global partners in communities with which they work to promote individual, structural and systemic change. They do this in a way that reflects the contextually derived and locally owned collaboration.	This learner has advocated for decolonial and anti-racist local leadership of programs and funding as they relate to coloniality, racism, and supremacy. They work to actively advance equity, decoloniality and antiracism as well as to actively dismantle structures that uphold the persistent oppression, racism and colonialism that marginalize those from resource-constrained or low/middle-income countries. They work to collaboratively and actively reimagine and rebuild the practice of global health through a justice-driven lens.

Educator Assessment of a Learner
