# Supplementary material for: Assessing content validity of learner milestones for decolonial global health education: A modified Delphi study
Source: PLOS Glob Public Health. 2026 Mar 17;6(3):e0005016. doi: 10.1371/journal.pgph.0005016 (PMC12994781; doi:10.1371/journal.pgph.0005016)
Supplement: S1 Text — (DOCX) [file pgph.0005016.s003.docx]

**S1 Text: Qualitative Data Excerpts**

Selected data from the Delphi participant feedback by round has been included below:

Round 1: Foundational Principles under the Critical Action Learner Level

1. There seem to be several dimensions of interest in this item, noting opportunity to measure different variables (could be 2 items)?

2. Suggest removing 'but' and 'not' negatives and refocus on what the learner can do/educator can assess ; also, consider that most often with action, either we do-don't on a scale & it's hard to measure what 'try' looks like.

3. Critical Action with intention & CURIOSITY is key to cultural humility as the process to support the outcome of cultural safety. There is another dimension of respecting how Critical Action requires earned Confidence thru SKILLFUL MEANS for decolonial and antiracist approaches. (I may have good intentions, but that may not necessarily play out into practice).

Suggest the following that incorporates Curious Intention as a foundation for Skillful Means and a decolonial strengths based approach that focuses on what the learner can do as an empowered change agent & lifelong learner. LEARNER: - (unique dimension ? - action)"I am actively using decolonial and anti-racist practices in my work (both personally and professionally)." - (unique dimension? -confidence for skillful means) In my efforts, I am confident in my ability to do this at the individual and interpersonal levels. -(unique dimension? lifelong learning with empowered community assuming that social innovation is inherently collaborative) I am continuing to learn how to apply these skills more broadly to affect structural change. I am curious to keep learning with others, and promote these antiracism and decoloniality skills and practices as a collaborative effort. EDUCATOR: - (unique dimension ? - action) This learner is actively trying to utilize decolonial and anti-racist practices in their work (both personally and professionally). - (unique dimension? -confidence for skillful means) They are confident in their ability to do this at the individual and interpersonal levels and engaged in lifelong learning to apply these skills more broadly to affect structural change. -(unique dimension? lifelong learning with empowered community assuming that social innovation is inherently collaborative) They are curious as to how to promote antiracism and decoloniality skills and practices in collaboration with others

Round 2: Leadership and Development Domain under the Pre-Contemplative Learner Level

I really appreciate the wording "I do not yet know how..." This shows promise to develop cultural humility & honestly reflect on their performance in 'leadership and development'. For the Educator assessment - it is inaccurate to state what the student thinks/considers - instead please revise with something like "The learner is able to recognize colonialism and needs to further analyze related persistent harms." AND instead of "they have not" (judgement) shift to "they have not yet" (learning process) AND the Educator Assessment could add recognition of a strength to build on - something like "the student has a demonstrated strength in their humility to recognize their emerging learning and skill development" in an effort to decolonizing evaluation, we need to be highly respectful and support a strengths-based approach to leverage challenges and promote growth. *see comments on Reflection & Transformative Learning theory (Domains 1 &2)

Round 3: Foundational Principles under the Contemplative Learner Level

Revise with congruent Behaviorist approach to Evaluation with assessment of observable behaviors AND positive reinforcement by the Educator. This requires minor but important changes to wording. With this in mind, suggest the following updates: Educator Assessment of Learner: This learner's actions convey curiosity as to how and why coloniality and racism exist and manifest themselves in global health. This is evidenced by how they realize that coloniality and racism are perpetuated by current global health practices and may wonder how to address this in their own work (*open ended with clear direction for behavioral change & positive reinforcement). The Learner is encouraged to be more curious as to how and why coloniality and racism exist and manifest themselves in global health.
